# Social support systems as determinants of self-management and quality of life of people with diabetes across Europe: study protocol for an observational study

**DOI:** 10.1186/1477-7525-12-29

**Published:** 2014-03-04

**Authors:** Jan Koetsenruijter, Jan van Lieshout, Ivaylo Vassilev, Mari Carmen Portillo, Manuel Serrano, Ingrid Knutsen, Poli Roukova, Christos Lionis, Elka Todorova, Christina Foss, Anne Rogers, Michel Wensing

**Affiliations:** 1 Radboud University Medical Centre, Scientific Institute for Quality in Healthcare, Nijmegen, The Netherlands; 2 Faculty of Health Sciences, NIHR Wessex CLAHRC, University of Southampton, Hampshire S017 1BJ, UK; 3 Department of Adult Nursing, University of Navarra, Navarra, Spain; 4 Education, Health and Society Foundation, Murcia, Spain; 5 Clinic of Social and Family Medicine, Faculty of Medicine, University of Crete, Voutes, P.C. 71003, P.O. BOX 2208, Heraklion, Greece; 6 Department of Economic Sociology, University of National and World Economy, Sofia, Bulgaria; 7 Institute for Health and Society, University of Oslo, P.O. Box 1130 Blindern, N-0318 Oslo, Norway

**Keywords:** Quality of life, Self-management, Chronic illness, Diabetes type 2, Social networks, Community organisations, Deprivation

## Abstract

**Background:**

Long-term conditions pose major challenges for healthcare systems. Optimizing self-management of people with long-term conditions is an important strategy to improve quality of life, health outcomes, patient experiences in healthcare, and the sustainability of healthcare systems. Much research on self-management focuses on individual competencies, while the social systems of support that facilitate self-management are underexplored. The presented study aims to explore the role of social systems of support for self-management and quality of life, focusing on the social networks of people with diabetes and community organisations that serve them.

**Methods:**

The protocol concerns a cross-sectional study in 18 geographic areas in six European countries, involving a total of 1800 individuals with diabetes and 900 representatives of community organisations. In each country, we include a deprived rural area, a deprived urban area, and an affluent urban area. Individuals are recruited through healthcare practices in the targeted areas. A patient questionnaire comprises measures for quality of life, self-management behaviours, social network and social support, as well as individual characteristics. A community organisations’ survey maps out interconnections between community and voluntary organisations that support patients with chronic illness and documents the scope of work of the different types of organisations. We first explore the structure of social networks of individuals and of community organisations. Then linkages between these social networks, self-management and quality of life will be examined, taking deprivation and other factors into account.

**Discussion:**

This study will provide insight into determinants of self-management and quality of life in individuals with diabetes, focusing on the role of social networks and community organisations.

## Introduction

### Background

Long-term conditions, such as diabetes and cardiovascular disease, pose major challenges for healthcare systems in economically developing and developed countries [1]. Diabetes type 2 is an increasingly prevalent condition with major impact on mortality, quality of life, and healthcare costs [2]. The prevalence of diabetes is rising as a consequence of ageing populations and unhealthy lifestyles. In the European Union, about 53 million adults aged 20–79 years had diabetes in 2013 with a predicted number of 64 million in 2030 [2]. People with low socioeconomic status [3] and in economically deprived areas [4] are at a higher risk of developing diabetes. Healthy lifestyles contribute to the prevention and improvement of this condition, while drug therapy is crucial for the prevention of long-term complications [5]. Therefore, optimizing self-management of people with diabetes (and many other long-term conditions) is an important strategy to improve health related quality of life and other outcomes, as well as improving the sustainability of healthcare systems. However, the effects of patient education and counselling on health-related life styles and adherence to treatment are mixed and the overall evidence for the effectiveness of such interventions is equivocal [6,7]. So the challenge is to optimize the reach and effectiveness of self-management support for people with long term conditions, particularly in vulnerable groups, such as people living in socially and economically deprived conditions [8]. Social participation and supportive social networks are increasingly recognized as important for illness management and may offer new perspectives for enhancing quality of life in people with chronic illness [9].

Self-management is a complex concept, which has been defined in different ways. We use the following definition: “*the care taken by individuals towards their own health and well-being: it comprises the actions they take to lead a healthy lifestyle; to meet their social, emotional and psychological needs; to care for their long-term condition; and to prevent further illness or accidents*” [10]. Self-management has been estimated as being beneficial for 70-80% of people with chronic conditions, and forms part of a wider agenda about public health, health promotion and patient involvement in different health systems across Europe [10]. Some effort has been made to identify groups that benefit most from self-management interventions. A study in the UK suggests that younger people and people with lower self-efficacy and health-related quality of life improve most by this type of interventions [11] and a Danish study shows that a low educational level hinders participation in self-management programs [12]. Literature also indicates that self-management interventions might be less attractive to males [13].

The current economic crisis in Europe has forced many governments to cut budgets for health expenditure. Self-management, which focuses on the patient taking the lead in the management of his or her condition, might offer a possibility to reduce use of healthcare services and thus costs. Likewise, social support for self-management might contribute to lowering of healthcare costs. Although both self-management and social support to improve self-management seem to be driven by societal need and also by ideology, scientific knowledge of the impact of social support and underlying influencing factors remains limited [14]. Some indication is given by a study in the UK suggesting that community and network-centred approaches may be particularly relevant for engaging people in socially and economically deprived areas [15]. Another study in the United Kingdom [16] explored social support systems of people with diabetes.

The study protocol presented here, as part of the EU-WISE project (EU-WISE is a research project funded by the EU’s Seventh Framework Programme), builds on this research and will examine the role of social support and networks in self-management for people with diabetes type 2 across Europe. The overall aim of the EU-WISE project is to provide better understanding of mechanisms involved in the management of diabetes, with a specific focus on socially disadvantaged people, on enhancing better self-management in peoples’ everyday lives and local communities, as well as on developing an understanding as to how this will work within different contexts. The EU-WISE project comprises a range of studies, using a mix of research methods. Literature studies on structure and governance of health and welfare systems, personal networks and community group networks will be done in the EU-WISE project as well as a qualitative and quantitative study. Finally, we will work on the development and assessment of an intervention based on the former studies. This study protocol concerns a quantitative survey study that is part of the EU-WISE project.

### Aims and objectives

**Figure 1:**
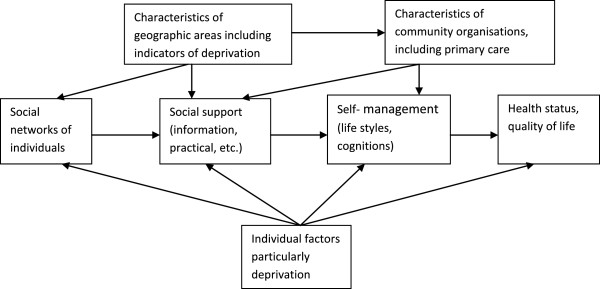
**Conceptual framework for the study.** Legend: Blocks refer to cluster of variables and arrows refer to expected causal effects.

The study has two overall aims: (1) to describe and explore the role of social networks in providing support to people with diabetes, (2) to describe and explore the role of community organisations (including healthcare providers in the community) which intend to support people with diabetes. The following overall objectives have been specified:

1. To describe the key aspects of the individual’s social network, social support and self-management in individuals with diabetes in six European countries, with a particular focus on people who are economically deprived or marginalised.

2. To describe the community organisations that support self-management in people with diabetes, and to map out the connections between these organisations.

3. To explore the associations between aspects of individuals’ social networks , affiliation with community organisations, self-management, health-related lifestyles, with a focus on individual’s quality of life and a special interest in the role of socioeconomic deprivation (as specified in Figure 1).

### Theoretical background

The role of social networks and community organisations for individual quality of life is mitigated by their role in health-related life styles, which are often described as self-management. Self-management of diabetes is demanding in many ways: it involves cognitive, practical and socio-emotional tasks. Drawing on personal capabilities, social networks, and the support available through the healthcare system, some people manage their diabetes well. For others, the capacity to self-manage is limited by medical problems, psychological factors, economic constraints, cultural influences, and lack of social capital [17]. While self-management has often been defined as an individually-centred concept, there is growing recognition of the need to consider contextual factors in the self-management of long-term conditions [15]. This notion is consistent with epidemiological research evidence. For instance, a systematic review found that the likelihood of survival was higher in people with stronger social relationships [18]. It is also illustrated by empirical studies, which show that the range of health-related behaviours are not randomly spread in a population, but linked to social network structures [19-21]. This has led to the notion of hypothesized “contagion processes” operating in social networks, which seem to apply to a range of items, including the spread of happiness, health-related behaviours, diseases and risk factors (e.g. smoking, obesity, and depression) [9,22]. The underlying mechanisms of contagion patterns are probably heterogeneous, depending on the item of interest. For behaviours, psychological mechanisms such as imitation of successful behaviours, role modelling and social comparison may explain contagion.

Different theoretical perspectives provide clues for identifying the relevant social system-related or contextual determinants of self-management in people with diabetes. Social-constructivist theory emphasizes that individuals develop ideas and behaviours in interactions with others, thus building realities that influence the frame of reference of individuals. This may suggest that self-management is influenced by social networks, regarded as “systems of support” or “communities of practice”. These include personal communities, community organisations, health professionals, and non-health professionals [23]. A realist review of studies suggests that social networks have a range of functions, including shaping of knowledge, discourses and narratives; shaping of stigma and deviance; negotiation of responsibilities and coordination; relationships with health services; and substitution of health professionals by lay networks [15]. Community organisations that provide support for people with long term conditions may be more responsive to social and environmental influences on condition management than traditional health services [24]. Network ties may operate through connections from patients to local organisations as part of a pathway of care as well as raise awareness of the group’s activities with other organisations through inter-organisation networks.

The concept of social capital can help to explain how social context influences self-management and quality of life in people with long term conditions. Social capital has been defined as an individual characteristic related to somebody’s networks, such as access to people or entities with relevant resources (e.g. information, practical help, access to medical care) [25]. Many survey studies use this individual-centred definition to explore relations between social capital and health outcomes in populations, without stating clear conclusions about the dynamics involved, due to lack of consistency in definition, measurement and plausible theories to explain the obtained correlations [26,27]. A further limitation is the predominance of individual (“ego”) networks rather than whole networks, that also include connections between friends or family members (“alters”).

Later definitions of social capital define it as a quality of social relationships or society at large, focusing on social trust that facilitates cooperation for mutual benefit [25]. In empirical research, this notion translates into analyses of the impact of the social capital of geographically defined neighbourhoods on their members’ health status [28]. The notion that social capital may be conceptualized as a feature of relations rather than individuals has some resonances with the sociological theory of the emergence and persistence of cooperation, although this focuses on specific network structures rather than using social systems metaphorically. This theory offers explanations for the development of cooperation in social systems, which imply a (risk of) loss for the decision- maker in the short-term compared to alternative behavioural options [29]. Altruistic behaviours, such as providing social support, can be seen as a special type of cooperation. The theory suggests, among other things, that cooperation is more likely in situations with (anticipation of) high likelihood of repeated contact and exchange (direct or indirect reciprocity), high density of connections (reducing “free rider” behaviours), as well as a physical location or position in a social network that is close to potential partners for collaboration.

A social network approach can thus make a positive contribution to applying the knowledge from social capital literature to the study of self-management and quality of life. In this research project, we will focus on a number of system-related determinants of self-management in people with diabetes. The empirical measures focus on the connections between individuals and between community organisations, which are relevant for receiving information on disease and management, practical help with daily tasks, or emotional support. Self-management behaviours, health-related life styles (smoking, physical exercise), quality of life and patient reported health status are outcomes of interest. In particular, the relevance of the following factors will be explored:

● Determinants with direct impact in the individual’s social network (“social capital factors”), such as ego-network size, number of connections with perceived high helpfulness, number of individuals in the wider network who have health-related knowledge, distance and frequency of contact with network members, diversity of types of relationships.

● Determinants indicating the impact of network structures (“contagion factors”), such as ego-network density, number of closed triads, homogeneity of the network in terms of age and gender.

● Determinants linked to individuals’ affiliations in the wider social system (“system factors”), such as the number of linkages of the individual to community organisations, density of connections between community organisations, deprivation of the neighbourhood.

To explore the effects of these determinants, we will study them across a wide range of countries, areas and individuals reflecting different levels of deprivation, urbanization and austerity policies.

In the study, we will also consider and (where possible) control for the influence of individual characteristics, such as age, gender, diabetes severity, co-morbidity and educational level.

Figure 1 provides a schematic global overview of the main domains (blocks) of factors in the study and their relationships (arrows), which will be explored in this research project.

## Methods

### Study design

An observational study in two related parts is planned: a cross-sectional observational study in individuals with diabetes (recruited through healthcare practices) and a survey of representatives of community organisations. The research will be conducted in 18 purposefully chosen geographic areas in 6 countries, which reflect a variety of healthcare systems: Bulgaria, Greece, the Netherlands, Norway, Spain and the UK. Thus, the study has a nested sampling design: individuals are nested in healthcare practices, both are nested in geographic areas, which are nested in countries. We plan to include community organisations, which are nested in the same geographic areas. The study is undertaken in six country-specific research teams, which have received approval from the countries’ relevant ethical committees to take part in the research. A full list of ethical committees approving this study can be found in Additional file 1.

### Setting

In each of the participating countries, research will be undertaken in three purposefully selected geographic areas: a deprived urban area (e.g. an area in a city); a relatively affluent urban area; and a deprived (relative to country) rural area. Urban is defined as located in a city with more than 100,000 inhabitants. Rural is defined as located in towns or villages with less than 30,000 inhabitants. We will use a high percentage of households with low household income as an indication for the socio-economic deprivation of a region. The affluent area has been included to explore the impact of geographical area on the outcomes. More specifically, we expect to find differences regarding the type and number of community organisations and levels of social trust between deprived and affluent areas. The rural area was included because people in those areas were expected to face different challenges in self-management behaviours.

In each country, these areas were chosen close to each other when feasible (the urban areas ideally in the same city) in order to get a relatively homogenous sample and thus some control for contextual factors (confounders related to area characteristics). There was no intention to get a representative set of areas for a larger region or country. In this way, we planned a study in 18 areas spread over 6 countries (ideally, clusters of 3 geographically closely located areas in each of 6 countries). From a statistical perspective, countries and geographical areas are considered ‘fixed’ (no statistical generalization beyond chosen areas and countries).

### Sampling of adults with diabetes

We plan to recruit a sample of 300 individuals in each country (100 in each area) with diabetes type 2. Inclusion criteria are: medical diagnosis of diabetes (not a patient reported diagnosis); type 2 diabetes only (no type 1, but comorbidities such as cardiovascular disease are allowed); age of 18 years or over. Exclusion criteria are: no established diagnosis of diabetes, but (e.g.) obesity or high risk for developing diabetes; mix of type 2 and type 1 (not pure type 2 diabetes); pregnancy; pregnancy-related diabetes; recent/current major surgery or medical procedures; severe cognitive or psychiatric handicap; terminal illness/receiving palliative care; absence of translators (e.g. family members) for patients with insufficient language skills.

Eligible patients will be recruited from healthcare practices (primary care practices in most countries) in the chosen geographical areas. Recruitment of individuals from primary care contexts is preferred because it has the advantage of a confirmed diagnosis of diabetes by a physician and provides the possibility of a face to face contact with the patient. This face to face contact, rather than just mailing a written or online questionnaire, is planned to enhance recruitment, especially for people from a deprived background [30].

Eligible patients will be given an invitation letter and a written questionnaire. The letter describes the study and the request to the patient to complete a written questionnaire and to be interviewed. Patients who give informed consent will be followed up by the researchers if they fail to complete interviews. The total number of individuals invited to participate will be recorded in order to calculate a participation rate.

### Sampling of community organisations

We plan to recruit up to 150 representatives of community and volunteering organisations per country (the number of organisations is probably lower than the number of representatives). The sample will consist of up to 150 respondents who will be purposefully selected to include community organisations that operate on the national, regional, and local level. The organisations will be selected, as much as possible, in the same geographical areas where the individuals with diabetes will be recruited. As some organisations do not operate in specific regions (e.g. webbased communities), we do not expect a total overlap between the areas where patients and organisations are recruited.

The recruited organisations will consist of community and volunteering organisations offering illness- relevant support to people with diabetes. Four main types of organisations are targeted: diabetes- related organisations; health- and healthy lifestyle-related organisations; well-being-related organisations; people’s and patients’ rights organisations. Diabetes related organisations are groups and organisations that have a direct focus on health improvement specifically on diabetes e.g. diabetes foundations and diabetes education organisations, associations and forums. Health- and healthy lifestyle-related organisations are groups and organisations that can improve health outcomes but do not explicitly focus on people with diabetes. These can include exercise-related organisations, diet groups and organisations for elderly people, which may have impact on self-management behaviours (e.g. walking groups). The third group refers to well-being-related organisations such as community centres where people meet and socialise (e.g. discussion circles). The fourth category of organisations consists of people’s and patients’ rights organisations that protect the position of patients. These could include for example advocacy groups for diabetes patients and elderly rights organisations. We will also include the healthcare providers, who provide access to individuals with diabetes for sampling, in the sample of community organisations.

To identify relevant community organisations we will adopt a bottom-up approach. The research teams in each country will start identifying a set of key organisations that are the most relevant within each type of organisations. Next, a combination of different approaches can be adopted with respect to the attributes of a specific country and area. These approaches are:

● Use the list of organisations suggested by other project partners and try to identify similar groups and organisations in each country.

● Use the information provided by one or more key persons knowledgeable about the areas where data will be collected e.g. a GP, a community centre, local council, etc.

● Include organisations that are mentioned in the patient’s interviews.

● Use the first group of organisation interviews to identify other organisations with the help of the snowballing procedure.

In each organisation, a representative may be an individual who is closely involved with the management of day to day operations, and/or the strategic development of the group/organisation. Larger organisations with independent groups in different areas, e.g. diabetes groups affiliated with Diabetes UK are seen as local branches. We will treat these as separate organisations and representatives of each of these groups can be interviewed separately. If the research team wants to interview two or more different representatives of the same local organisation this will be allowed. The purpose of this would be to get more reliable data on the key links of the organisation, which will increase the validity of the information from the surveys (less likely to be useful for smaller organisations).

### Statistical accuracy

The planned study will include diabetes patients (n = 1800), primary care practices (n = 36 to n = 96), support organisations (n = 300 to 900), geographical areas (n = 18) and countries (n = 6). To assess the statistical accuracy of the associations between aspects of individuals’ social networks and support, affiliation with community organisations, self-management and health status a tentative power analysis was done. Based on α = 0.05, power = 0.80 and the inclusion of eight independent variables in the analysis the sample size will allow the detection of a medium effect size (*f*^2^ = 0.15) [31]. Because of the clustering of patients within areas (reflecting both country differences as well as primary care practices differences and regional differences), we took the design effect into account. Between-practice variation for aspects of patients’ health status or behaviours tend to be low compared to measures of healthcare delivery [32]. A study on diabetes patients in primary care practices showed on most outcome measures an intraclass correlation coefficient (ICC) < 0.05 [33]. Relevant outcome measures such as the SDSCA and SF-12 showed an ICC of 0.022 and 0.028. We therefore assume an ICC of 0.03. The design effect is calculated as *DE* = 1 + (m-1) ρ, with 100 patients per cluster, resulting in design effect of 3.97 and an effective sample size of 1800/3.97 = 450 patients. This effective sample size is sufficiently large to detect a medium effect size.

### Patient questionnaire measures

The study uses a pre-structured patient questionnaire, which utilizes both established and purposefully constructed measures in order to explore a range of domains. The questionnaire has two parts. The first part includes a written questionnaire with demographic variables quality of life items, selfcare, received care and participation in local organisations. The second part is a face-to-face or telephone interview, which will provide information on the social networks and support of the respondents. When available, we use measures that have been translated into relevant languages, validated in several health systems, provide reference data (for comparison), and shown to be feasible in people with low education (thus, short and simple). The source-versions of the questionnaires are available in English. If no validated translation into country-specific languages is available, a structured procedure for translation, involving forward and backward translations is applied. Table 1 provides an overview of the measures included in the patient questionnaire.

**Table 1:** Overview of measures in patient questionnaire (English versions)

**Measure**	**Concept**	**Number of items**	**Link**
*Outcome measures*			
SF-12v2 4-week recall	Functional health status	12	http://www.qualitymetric.com/WhatWeDo/SFHealthSurveys/SF12v2HealthSurvey/tabid/186/Default.aspx
European social survey	Well-being	2	http://www.europeansocialsurvey.org/
Rapid assessment of physical activity	Physical activity	9	http://depts.washington.edu/hprc/rapa
The summary of diabetes self-care activities	Selfcare behaviour and life style	12	http://care.diabetesjournals.org/content/23/7/943.full.pdf
Morisky medication adherence scale	Medication adherence	4	https://www.gem-beta.org/public/MeasureDetail.aspx?mid=1133&cat=2
HEIQ V3.0; self monitoring and insight	Selfcare cognitions	6	http://www.deakin.edu.au/health/research/phi/heiQ.php
HEIQ V3.0; skill and technique acquisition	Selfcare cognitions	4	http://www.deakin.edu.au/health/research/phi/heiQ.php
*Inter-mediate measures*			
Diabetes Health Care Utilization questionnaire	Received medical and social care	5	http://patienteducation.stanford.edu/research/utilizdiabetes.html
Age, sex, family situation, education, employment status, sick leave, ethnicity, housing, global household income and comorbidities	Demographic data	14	
*Independent measures*			
Involvement in regional or national support organisations		2	
Name generator using probes	Network members delivering support	3	
Pre-defined broad domains: information, treatment, day to day tasks, emotional support	Types of delivered support by network members	3	
Gender, age, and type of connection	Network members characteristics	6	
Relations between network members	Ego-network	1	

#### Written questionnaire

As outcome measures we will measure both individual health status as well as physical lifestyle. To measure functional health status we will use the SF-12 with 4-week recall. This a patient reported health status measure developed to measure the disease burden, both physically as mentally [34]. Besides health status we also measure health-related well-being, using two items from the European Social Survey which measures happiness and life satisfaction (http://www.europeansocialsurvey.org).

To assess physical life style of respondents, the Rapid Assessment of Physical Activity (RAPA) is used to measure physical life style of respondents. This questionnaire was developed to measure the level of physical activity of older patients [35]. The Summary of Diabetes Self-Care Activities (SDSCA) assesses selfcare behaviour and life style because selfcare for diabetes patients is closely related to life style. The SDSCA measures behaviours such as diet, smoking, physical exercise, blood sugar testing and foot care [36]. Medication adherence as a selfcare behaviour is assessed using the Morisky Medication Adherence Scale (MMAS-4). This questionnaire measures both medication adherence as well as barriers for medication adherence [37]. Selfcare cognitions are measured by two domains from the HEIQ V3.0: the self monitoring and insight domain and the skill and technique acquisition domain. The former assesses the ability of patients to measure their condition and their insight in performing selfcare. The latter captures the patient’s knowledge and ability to perform the actions to relieve the disease symptoms [38].

As intermediate variables we retrieve data on the medical and social care received in the past six months with the use of the Diabetes Health Care Utilization questionnaire. This questionnaire is developed to measure health care utilization by a self reported list [39]. Furthermore we collect demographic data, including patients’ age, sex, family situation, education, employment status, sick leave, ethnicity, housing, global household income and comorbidities. In order to map out affiliation networks we also measure involvement in regional or national support organisations.

#### Interview

In interviews with patients data on their social network and social support will be collected. First, the name generator method [40] is used to generate a list of relevant individuals and using probes for family members; friends, neighbours, colleagues; and professional care providers. Next, for each listed individual we will collect a number of characteristics, including gender, age, type of connection and the received support according to pre-defined domains: information, treatment, day to day tasks, and emotional support. From the named individuals (“alters”) the perceived connections between each individual will be listed as this is crucial for mapping out the complete ego-network. Finally, the position generator is used to identify access to people with specified healthcare professions. All questions have been tested before data collection started using cognitive testing techniques.

### Community organisation questionnaire measure

A telephone or face-to-face survey will be conducted with individuals who represent a support organisation. The questionnaire is purposefully developed and covers the following domains: descriptive information on the organisation and its activities; reach in target group in terms of users of information, participants in activities; collaboration with other support organisations in the local area, including primary care healthcare practices; contact/collaboration with other organisations in domains that are relevant to self-management behaviours.

### Measures concerning primary care practices and geographic areas

At a higher organisational level we will collect data on the characteristics of healthcare practices, geographic areas, support organisations, and contexts from which patients are recruited. Concerning each practice we will collect information about the practice size in terms of number of patients and staffing e.g. number of physicians, nurses, and assistants. In primary care practices, will collect some items about the practice organisation. Concerning each geographic area we will collect some descriptive information such as the urban/rural nature, deprivation, number of inhabitants and age structure.

### Data analysis

Data collected in different countries will be checked for integrity and then included into a comprehensive database, which will be finalized prior to data analysis. In the first phase of the analysis, the characteristics of individuals and organisations will be described, including the social networks. Scale scores and network measures will be constructed in this phase. This provides answers to research questions 1 and 2. The second phase of the analysis addresses research question 3 and comprises an exploration of linkages between system-related factors (in social networks and community organisations) on the one hand, and self-management, health-related lifestyles and quality of life on the other hand, taking deprivation and other factors into account (Figure 1).

To explore the relevance of system-related factors for patients’ self-management and other outcomes (research question 3) we will develop and test a number of hypotheses. First we will explore determinants based on the idea of social capital. We expect that more social capital will result in better self-management and a higher quality of life. Relevant determinants for social capital are: ego-network size, number of connections with perceived helpfulness, number of individuals in the wider network who have health-related knowledge. Second, we will explore the role of contagion in social network structures. We expect individuals to adopt behaviour from other network members more often if a network has a higher density, more closed triads and a higher homogeneity in terms of age and gender. The third perspective takes the wider social system into account. We expect that more individual embeddedness into community organisations will result in better self-management and a higher quality of life. Moreover, we expect that a higher density of connections between community organisations and a lower deprivation of the neighbourhood will lead to better self-management and a higher quality of life in individuals.

In all analyses, we will consider a range of other factors including age, gender, diabetes severity, co-morbidity and educational background. In particular, we will examine whether the main effects (e.g. of social support and community organisation on self-management behaviours) are moderated by deprivation levels.

Network characteristics will be calculated using specific social network analysis software. For other analyses we will use multilevel regression models, taking clustering on the level of country, area and healthcare provider into account. To reduce the possibility of chance capitalization, we will use p < 0.05 in hypothesis-driven analyses to indicate significance, but in explorative analyses we will use p < 0.01. Testing differences between countries is not planned as the sampling method does not allow inference to countries, but we will take country differences into account when interpreting the results.

## Discussion

The current economic crisis in Europe has forced many governments to cut budgets for health. Self management is seen as one possible way reduce costs, forcing the patient to take the lead in his/her health and shifting social support towards family and community organisations. This implies that social support is expected to be more often delivered by family members and community organisations and stimulating them to take on new areas such as support for self- management. Some research on the role of social support and community organisations has been done, suggesting that community and network-centred approaches may be particularly relevant for engaging people in socially and economically deprived conditions [15]. We will explore the effect of social capital factors, contagion factors and system factors on self-management and quality of life. Thus the study provides a systems perspective on how individuals with chronic illness use self-management to improve their health and quality of life. To explore the effects of these determinants, we will study them across a wide range of countries, areas and individuals, reflecting different levels of deprivation, urbanization and severity of austerity policies.

The social network approach of this study is likely to make a contribution to applying the knowledge from social capital literature to the study of self-management support. Moreover, the wide range of settings can provide us a better understanding how self-management and social support will work within different contexts. Finally, we will provide insight into the potentially moderating influence of social networks and social support on the negative impacts of deprivation on self-management and health-related behaviours.

## Competing interests

The author(s) declare that they have no competing interests.

## Authors’ contributions

JK and MW are responsible for the design of the study, development of the questionnaires and provided the first draft of the manuscript. AR, MW, IV wrote the original study proposal for the overall EU-WISE project. All authors provided input in plenary meetings regarding the design of the study and commented on the final manuscript. All authors read and approved the final manuscript.

## Supplementary Material

Additional file 1 Ethical approval per country. Click here for file
