# Supplementary material for: Social support systems as determinants of self-management and quality of life of people with diabetes across Europe: study protocol for an observational study
Source: Health Qual Life Outcomes. 2014 Mar 4;12:29. doi: 10.1186/1477-7525-12-29 (PMC4021429; doi:10.1186/1477-7525-12-29)
Supplement: Additional file 1 — Ethical approval per country. [file 1477-7525-12-29-S1.docx]

**Ethical approval per country**

| **Country** | **Approval** |
| --- | --- |
| Bulgaria | Ethical approval by UNWE and the NCPHA (National Center for Public Health and Analysis). |
| Greece | The research team of the University of Crete has received the Bio-ethical Approval by the Scientific & Bio-ethical Committee and the Administration Council of the Regional Academic Hospital (PAGNI) of Heraklion Crete (*Date: 27/06-12-2012, No. 1087- 644/27/31-7-2012, Decision 974/26/20-11-2012).* |
| Netherlands | Ethical aprroval by CMO region Arnhem Nijmegen, number 2013/098 |
| Norway | REK 2012/593b and OUS-HF 2013/2922 |
| Spain | Obtained the ethical approval from Ethics Commission of the University of Navarra. Reference number: 014-2013 |
| Uk | Covered under decisions by University of Manchester Research Ethics Committee, November 2009 (ref. 09130); Greater Manchester Research Ethics Committee, February 2010 (ref. 10/H1008/1); Greater Manchester Research Ethics Committee, July 2012 (ref. 10/H1008/1 amendment 2),Salford and Trafford local research ethics committee (ref. 09/H1004/6), University of Southampton Ethics and Research Governance Online, March 2013 (ref. 4340). |
